# The gift of preexisting immunity for developing an alternative vaccine strategy

**DOI:** 10.1172/JCI174952

**Published:** 2023-12-01

**Authors:** Kim A. Tran, Maziar Divangahi

**Affiliations:** 1 Meakins-Christie Laboratories, Department of Medicine, Department of Pathology, McGill University Health Centre, Montreal, Quebec, Canada.; 2 McGill International TB Centre, Montreal, Quebec, Canada.

## Abstract

Despite the worldwide application of vaccination and other antiviral interventions, pulmonary viral infections remain a persistent threat to human health. The 1918 influenza pandemic killed more than 40 million people in just one year, and the SARS-CoV-2 pandemic has killed more than 6.9 million people since 2019. While the current approved COVID-19 vaccines are administered parenterally and induce systemic immunity, they only prevent the progression to severe disease. Thus, other vaccine platforms are still needed for completely preventing the disease and subsequent transmission. In this issue of the *JCI*, Kawai et al. present an adjuvant-free subunit (RBD-HA) fusion vaccine, which produces robust IgG and IgA antibody responses against SARS-CoV-2, enriched within the nasal cavity, by using the host’s preexisting immunity to influenza infection. This preclinical study has tremendous implications for future mucosal vaccine design and provides a roadmap for generating a safer and effective intranasal vaccine against pulmonary infections.

## Mucosal vaccination against COVID-19

**Figure 1:**
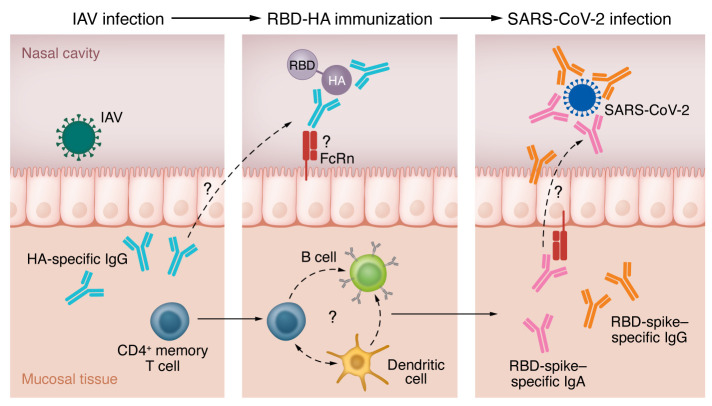
RBD-HA immunizes IAV-infected mice against SARS-CoV-2. Previous exposure to IAV infection results in the generation of specific antibodies to the immunodominant glycoprotein HA and activation of CD4^+^ memory T cells. Upon immunization with RBD-HA vaccine, circulating HA-specific IgG antibodies can cross the mucosal barrier and recognize HA antigens in the nasal cavity. Only HA-IgG–bound particles will have the capacity to cross the mucosal barrier potentially through transport via FcRn. Bound antigens will enter into the mucosa-associated tissue that is rich with immune cells (B cells, DCs, CD4^+^ memory T cells) for antigen presentation. The synergistic action of immune cells in the mucosal tissue will generate RBD-spike–specific IgG and IgA antibodies, which can cross into the nasal cavity and block viral entry during subsequent SARS-CoV-2 infection.

The SARS-CoV-2 pandemic brought an immense advancement in vaccine development, resulting in the worldwide distribution of mRNA vaccines. Although the current vaccines against COVID-19 are highly effective in preventing severe disease, they do not protect against infection and consequently still pose a risk of onward transmission. COVID-19 vaccines are administered parenterally and thus elicit robust systemic immune responses, but they generate poor immunity at the respiratory mucosa (1–3). As the nasal cavity is the site of viral entry, the generation of mucosal immunity in the upper respiratory tract would offer a substantial benefit of preventing breakthrough infection with the potential of sterilizing immunity and reduced viral transmission. Therefore, the use of mucosal vaccines to induce a localized immune response at the site of infection is an attractive tool for preventing the dissemination of emergent respiratory viruses. However, there are many challenges for generating mucosal vaccines. Mucosal tissues are continuously exposed to different microorganisms and have thus adapted multiple layers of barrier protection, including physical factors such as mucins, immune factors such as IgA antibodies, and secreted factors, including lysozymes and proteolytic enzymes (4). Thus, subunit vaccines formulated with pathogen-derived antigens are typically degraded within the mucosal lumen and cannot cross the mucosal barrier to generate a protective immune response. Furthermore, the development of mucosal subunit vaccines has stumbled due to the lack of safe adjuvants, as there are concerns about their side effects, including Bell’s palsy (5–7). In this issue of the *JCI*, Kawai and coauthors report on their generation of an adjuvant-free vaccine containing the receptor-binding domain (RBD) of the SARS-CoV-2 spike protein fused with the hemagglutinin (HA) glycoprotein derived from influenza A virus (IAV) (8). The authors hypothesized that previous exposure to influenza virus would ensure the host possessed HA-specific IgG antibodies, which can bind the RBD-HA antigen to cross the mucosal barrier, presumably via neonatal Fc receptor (FcRn), for efficient antigen delivery to B cells and DCs. They elegantly show that the intranasal administration of the RBD-HA vaccine in mice that were previously infected with IAV provided marked protection against SARS-CoV-2 infection. Importantly, the preexisting immunity to IAV was a key in generating neutralizing RBD-specific IgA and IgG antibodies mucosally and systemically against SARS-CoV-2 (8) (Figure 1).

## RBD-HA immunization generates robust antibody protection against COVID-19

The authors employed several vaccination methods to test the efficacy of their fusion vaccine: RBD-HA administered i.n., RBD plus the adjuvant c-di-GMP administered i.n., and RBD plus the adjuvant alum administered s.c. Following 30 and 51 days of IAV infection, the mice were immunized with a prime/boost vaccine strategy, respectively. RBD-specific antibodies were only detected in mice that were previously infected with IAV (IAV-mice). Strikingly, naive mice showed no detectable levels of RBD-specific antibodies, demonstrating that preexisting immunity to HA was required in this vaccine-delivery system. Interestingly, the level of systemic (i.e., plasma) RBD-specific IgG was comparable between IAV-mice immunized with RBD-HA and mice immunized with RBD plus alum (s.c.). Importantly, the level of local (nasal) RBD-specific IgA was also comparable between RBD-HA immunized IAV-mice and RBD c-di-GMP–immunized (i.n.) mice. These data collectively indicate that the generation of both systemic and mucosal antibody-specific responses was robust in RBD-HA–immunized IAV-mice. The induction of a potent IgA antibody response in the nasal cavity represents the major advantage of a mucosal vaccine, considering several studies have shown an inverse correlation of mucosal IgA concentrations with the risk of breakthrough SARS-CoV-2 infections (9–11). Thus, the generation of mucosal RBD-specific IgA can substantially prevent viral dissemination across the respiratory mucosal barrier.

The authors then isolated the antibodies from nasal wash alone and demonstrated that the antibodies were sufficient to neutralize different strains of SARS-CoV-2 in vitro by utilizing pseudotyped viruses displaying Alpha, Delta, and Omicron spike protein. To translate these observations in vivo, they next demonstrated that the nasal RBD-HA immunization in IAV-mice remarkably reduced morbidity and mortality as well as pulmonary viral replication against mouse-adapted SARS-CoV-2 infection. Interestingly, although the authors used an immunization volume that limited the immune response of RBD-HA to the nasal cavity, IAV-mice vaccinated with RBD-HA were also protected against infection in the lower respiratory tract. These data suggest that the protection mediated by RBD-HA in the upper airways prevents viral entry into the lower airways, demonstrating the advantage of mucosal immunization against breakthrough infection rather than only mitigating severe disease. This protection was mediated via the neutralization capacity of RBD-specific antibodies, as the passive transfer of serum from IAV-mice immunized with RBD-HA was capable of mediating protection from lower respiratory tract infection.

## The gift of preexisting immunity

A major hurdle for subunit mucosal vaccines lies in the delivery of antigen across the mucosal barrier for subsequent antigen presentation. Dynamic mucosal host defenses can degrade unadjuvanted antigens before even reaching the physical barrier of the respiratory epithelium (12). Kawai et al. (8) circumvented this obstacle, efficiently delivering peptide delivery across the mucosal barrier to antigen-presenting cells (APCs). They initially showed that HA-specific IgG antibodies were required for generating adequate RBD-specific antibodies, as coadministration of RBD-HA with competing HA yielded lower RBD-specific IgG. Importantly, the presence of IAV-IgG in the blood and nasal cavity was required at the time of RBD-HA immunization. In contrast, by using IgA^–/–^ mice, the authors showed that IAV-IgA was not necessary in the generation of RBD-specific antibodies following RBD-HA administration. Interestingly, preexisting memory CD4^+^ T cells were also required, as CD4^+^ T cell–depleting treatment abrogated the generation of RBD-specific antibodies in IAV-mice immunized with RBD-HA. Although these T cells could have been IAV-specific CD4^+^ memory T cells, prior i.p. administration of IAV-IgG was sufficient to simulate IAV preexisting immunity, which raises a question about the role of bystander CD4^+^ memory T cells in this process. These findings align with studies reporting an association between effector CD4^+^ memory T cells and antibody production in COVID-19 convalescent patients (13). Although the synergistic action of APCs, local and systemic IgG, and memory CD4^+^ T cells is required for RBD-HA immunization in IAV-mice, the crosstalk of this axis is incompletely understood.

## A translational advantage to an RBD-HA subunit vaccine platform

Kawai and colleagues present the efficacy of their vaccine only in a preclinical study, but this study shows great promise for future translational studies. First, the authors show their immunization model works in both BALB/c and C57BL/6 mice. Second, they provide evidence for the longevity of their immunization approach, showing RBD-specific antibodies were maintained up to six months after immunization. Third, to replicate a diverse history of infections seen in humans, Kawai et al. also utilized two common pulmonary pathogens, *Mycoplasma pneumoniae* and respiratory syncytial virus (RSV). They show that sequential infection with IAV did not alter levels of RBD-specific antibodies following RBD-HA. This result implies that infection history does not affect the efficacy of the RBD-HA vaccine as long as individuals have been exposed to IAV. In the case of countries with low IAV prevalence, the authors show that HA-specific antibodies are sufficient to mimic preexisting immunity. Fourth, the authors demonstrate that preimmunization with the COVID-19 mRNA vaccine can also promote the generation of RBD-specific antibodies. This finding suggests that preexisting immunity to SARS-CoV-2, in the context of immunization and likely infection as well, could be harnessed for the efficient action of an RBD-HA vaccine. Fifth, Kawai et al. show the versatility of their vaccine platform by generating HA fusion vaccines with the N-terminal of SARS-CoV-2 spike protein, the pneumococcal surface protein A of *Streptococcus pneumoniae*, and the central domain of surface G glycoprotein from RSV. With each fusion vaccine, specific antibodies for the derived antigen were generated and capable of providing protection against subsequent infection. These exciting findings uncover a promising avenue for subunit mucosal vaccines (8). In fact, mucosal sites cover a surface area up to 30–40m^2^ in humans and represent substantial entry points for different pathogens (14). This vaccine platform could be utilized against emergent respiratory pathogens, as the authors intended, but could also be explored to combat pathogens of other mucosal sites, such as common enteric pathogens and sexually transmitted diseases (STDs) that target the urogenital mucosal barrier.

## Conclusions

Emergent strains of SARS-CoV-2 and other pulmonary pathogens are in constant circulation, underscoring the necessity for preventative strategies that curb transmission. The tropism of pulmonary viruses for the upper versus lower respiratory tract dictates the severity of infection. For instance, highly glycosylated seasonal influenza viruses are often eliminated from the airways via the ciliary ladder, which causes a mild disease by limiting the infection to the upper airways. In contrast, the poorly glycosylated 1918 pandemic strain bypasses the upper airways, causing deep lung infection with severe disease (15). Thus, developing an effective mucosal vaccine that limits the infection to upper airways is a game changer. Although the exact cellular and molecular mechanisms remain to be determined, here Kawai et al. (8) provide a promising subunit vaccine platform that harnesses our preexisting immunity to generate neutralizing antibodies in the upper airways and can potentially be applied to diverse pathogens. Collectively, mucosal vaccines offer notable benefits of generating local and systemic immunity. Optimizing the development of such tools may represent an unprecedented advancement in vaccine design.

## Version 1 12/01/2023

Electronic publication
